# Genetic tests for estimating dairy breed proportion and parentage assignment in East African crossbred cattle

**DOI:** 10.1186/s12711-017-0342-1

**Published:** 2017-09-12

**Authors:** Eva M. Strucken, Hawlader A. Al-Mamun, Cecilia Esquivelzeta-Rabell, Cedric Gondro, Okeyo A. Mwai, John P. Gibson

**Affiliations:** 1 School of Environmental and Rural Science, University of New England, Armidale, 2350 Australia; 2 Pic Improvement Company (PIC), Genetic Services, Hendersonville, TN 37075 USA; 3 Michigan State University, Animal Science, East Lansing, Michigan 48824 USA; 4 International Livestock Research Institute, Nairobi, Kenya

## Abstract

**Background:**

Smallholder dairy farming in much of the developing world is based on the use of crossbred cows that combine local adaptation traits of indigenous breeds with high milk yield potential of exotic dairy breeds. Pedigree recording is rare in such systems which means that it is impossible to make informed breeding decisions. High-density single nucleotide polymorphism (SNP) assays allow accurate estimation of breed composition and parentage assignment but are too expensive for routine application. Our aim was to determine the level of accuracy achieved with low-density SNP assays.

**Methods:**

We constructed subsets of 100 to 1500 SNPs from the 735k-SNP Illumina panel by selecting: (a) on high minor allele frequencies (MAF) in a crossbred population; (b) on large differences in allele frequency between ancestral breeds; (c) at random; or (d) with a differential evolution algorithm. These panels were tested on a dataset of 1933 crossbred dairy cattle from Kenya/Uganda and on crossbred populations from Ethiopia (N = 545) and Tanzania (N = 462). Dairy breed proportions were estimated by using the ADMIXTURE program, a regression approach, and SNP-best linear unbiased prediction, and tested against estimates obtained by ADMIXTURE based on the 735k-SNP panel. Performance for parentage assignment was based on opposing homozygotes which were used to calculate the separation value (*sv*) between true and false assignments.

**Results:**

Panels of SNPs based on the largest differences in allele frequency between European dairy breeds and a combined Nelore/N’Dama population gave the best predictions of dairy breed proportion (r^2^ = 0.962 to 0.994 for 100 to 1500 SNPs) with an average absolute bias of 0.026. Panels of SNPs based on the highest MAF in the crossbred population (Kenya/Uganda) gave the most accurate parentage assignments (*sv* = −1 to 15 for 100 to 1500 SNPs).

**Conclusions:**

Due to the different required properties of SNPs, panels that did well for breed composition did poorly for parentage assignment and vice versa. A combined panel of 400 SNPs was not able to assign parentages correctly, thus we recommend the use of 200 SNPs either for breed proportion prediction or parentage assignment, independently.

**Electronic supplementary material:**

The online version of this article (doi:10.1186/s12711-017-0342-1) contains supplementary material, which is available to authorized users.

## Background

Based on bovine remains and terracotta figurines, it is assumed that the first domesticated cattle in Africa, around 5000 years ago, were humpless (*Bos taurus*) [1, 2]. Nowadays, the West African N’Dama cattle (*Bos taurus*) and closely related populations in West Africa are believed to be the only surviving population from the originally domesticated African cattle. Humped Zebu cattle (*Bos indicus*) were introduced to Africa with traders from Arabia 2000 to 3000 years ago [2, 3]. Crossbreeding of local African taurine with introduced indicine cattle created a variety of new populations that make up most of the native cattle of Africa today [4–6]. Based on analyses of karyotypes and genetic markers, Frisch et al. [7] inferred that East African Zebu breeds are a mixture of *Bos indicus* and *Bos taurus*, and that Sanga breeds are *Bos taurus*. Subsequent studies using microsatellites and then single nucleotide polymorphisms (SNPs) confirmed the mixed ancestry of East African Zebu breeds but identified that the *Bos taurus* component is primarily African rather than European *Bos taurus* [8]. Hanotte et al. [8] also found that the ancestry of the tested Sanga breeds was also mixed but with substantially higher proportions of African *Bos taurus* than Zebu breeds.

During the second half of the twentieth century, globalization and an increasing demand for milk fostered a new wave of crossbreeding in some parts of Africa. Northern American and European *Bos taurus* dairy breeds, known for their high production levels, were imported and crossed to native breeds in an attempt to improve the level of milk production. For example, in Kenya, Ayrshire, Jersey, and Guernsey cattle were originally imported, then Friesian and later Holstein dominated bovine imports. In Uganda, imports of Friesian and later Holstein cattle dominated [9]. The rapid and large-scale expansion of the East African highland dairy smallholders indicates that, under appropriate conditions, crossbreeding and the use of crossbred cattle can yield significant increases in smallholder income.

Knowledge of breed composition is required to determine which crossbreeds perform best under the wide variety of smallholder dairy systems, and also, to make breeding decisions for producing progeny of the desired breed composition. Because of the lack of pedigree records, the breed composition of most animals is not known [10]. Furthermore, the lack of knowledge about breed proportions and about the relationships within and between populations may lead to the loss of native genetic resources and may build-up inbreeding depression [11, 12].

High-density (HD) SNPs can be used to assess the levels of genetic diversity between individuals [13], to determine coefficients of kinship between pairs of individuals allowing for parentage exclusion [14], to obtain accurate estimates of breed proportions in crossbred animals [15], and to trace animal products to their source [16]. The HD SNP panels are too expensive for routine use in smallholder systems. Genotyping a few hundred SNPs can be relatively inexpensive but how accurate are the estimates of breed composition or parentage assignment when using such small numbers of SNPs in crossbred dairy populations is not known.

The aim of this study was to determine the accuracy and bias when using small subsets of SNPs from a commercially available 735k-SNP panel to estimate breed proportion and parentage assignment in crossbred dairy cattle populations in East Africa. We used a variety of methods to select the SNPs for reaching the highest possible accuracy (r^2^) of estimated breed proportions and parentage assignment. Based on the history of crossbreeding in Africa, we included as baseline information the genotype frequencies in pure breeds such as the N’Dama (reference for African *Bos taurus*), Nelore (reference for pure *Bos indicus*), and several European and North American dairy breeds, which collectively represent the ancient and more recent ancestral gene pool of the crossbred dairy animals.

## Methods

### Animals

In total, 1933 crossbred dairy cows and local indigenous breeds of Ankole (n = 43), Nganda (n = 17), and Small East African Zebu (Zebu; n = 58) were sampled from 845 households that are distributed at five sites in Kenya and two sites in Uganda (Dairy Genetics East Africa, DGEA1, project). In addition, genotype datasets for N’Dama (as the reference African *Bos taurus* breed; n = 20), Nelore (as the reference *Bos indicus* breed; n = 20), Guernsey (n = 20), Holstein (n = 20), and Jersey (n = 20) were sourced from the International Bovine HapMap consortium. Furthermore, British Friesian (n = 25) from the SRUC in Scotland and Canadian Ayrshire (n = 20) from the Canadian Dairy Network (CDN) were used as reference breeds.

An independent population of 545 crossbred animals from Ethiopia (DGEA2 project) was sampled from 400 households at nine sites. Instead of the Kenyan and Ugandan indigenous breeds, we included the Ethiopian Begait Barka (n = 30), Danakil Harar (n = 30), Fogera (n = 29), and Boran (n = 30) in the analyses of breed composition. An independent Tanzanian dataset (DGEA2 project) consisted of 462 crossbred animals sampled from 326 households at three sites. Tanzanian indigenous breeds for the analysis of breed composition included Iringa Red (n = 13), Singida White (n = 22), and Tanzanian Boran (n = 20).

### Genotype data

All animals were genotyped with the 777k-SNP BovineHD Beadchip (Illumina Inc., San Diego). In order to keep potentially interesting SNPs that could be excluded due to population stratification, criteria for genotype data filtering were applied per breed and focused on genotyping quality. Genotypes of the DGEA1 and 2 and SRUC data were filtered using ‘SNPQC’ an R pipeline for quality control of Illumina SNP genotyping array data described in [17] to eliminate SNPs that had a median GC score lower than 0.6 and a sample-wise call rate lower than 90%. Only the SNPs on the 29 autosomal bovine chromosomes were included in the analysis. Genotypes provided by the Bovine HapMap consortium and the Canadian Dairy Network were already quality-controlled. The cleaned population datasets were merged and included 735,293 SNPs. SNPs that were excluded after quality control in one breed but not in another breed were set to “not available” (NA) in the breed for which they were excluded.

We checked the relationships between animals based on the genomic relationship matrix [18], with missing genotypes being replaced by the average genotype (encoded as 0, 1, 2) across all animals:

$${\mathbf{GRM}} = {\mathbf{ZZ}}^{{\prime }} /2 *\sum {\text{p}}_{l} *\left( {1 - {\text{p}}_{l} } \right),$$

where ${\mathbf{Z}}$ is the centered genotype matrix and ${\text{p}}$ is the allele frequency at locus $l$. Matrix ${\mathbf{Z}}$ was constructed by subtracting from the genotype matrix ${\mathbf{M}}$ the ${\mathbf{P}}$ matrix, which equaled 2*(p − 0.5). The centering of ${\mathbf{Z}}$ was achieved by subtracting −1 from ${\mathbf{M}}$.

Inbreeding coefficients (*F*_IS_) were calculated per breed according to Weir and Cockerham [19].

### Observed breed compositions

Breed proportions of crossbred animals from both crossbred populations were estimated by using the full quality-controlled data in the ADMIXTURE 1.23 program [20]. Analyses were performed by assuming that N’Dama, Nelore, Ayrshire, Friesian, Guernsey, Holstein, and Jersey represented ancestral populations. We used all 735k SNPs to estimate breed proportions to create a baseline for comparison with the estimates using subsets of SNPs. Dairy proportion was defined as the sum of breed proportions across all European dairy breeds that was estimated in the crossbred populations. The Kenyan/Ugandan dataset also included the local pure breeds of Ankole, Nganda, and Zebu whereas the Ethiopian dataset included Begait Barka, Danakil Harar, Fogera, and Ethiopian Boran, and the Tanzanian dataset included Iringa Red, Singida White, and Tanzanian Boran.

### Observed pedigree

The pedigrees of the crossbred animals from Kenya/Uganda, Ethiopia, and Tanzania were reconstructed based on the presence or absence of opposing homozygotes [21, 22]. Opposing homozygotes (*opH*) occur if at the same SNP, two individuals carry opposite homozygous genotypes [21]. The more *opH* are found between two individuals, the less likely are these individuals related. Except for genotyping errors and mutations, a parent and offspring cannot display *opH*. The distribution of *opH* that are associated with parent–offspring or other relationships is specific to the allele frequencies of the population and the number of SNPs used; however, with several tens of thousands SNPs or more, parent–offspring relationships can always be clearly separated from other relationships. By applying the approach of Strucken et al. [23] if there are less than 1000 *opH*, it is possible to unambiguously distinguish between parent–offspring and unrelated individual pairs in the DGEA1 and 2 crossbred populations.

The Kenyan/Ugandan crossbred population contained 171 cows with 189 offspring, of which 15 cows had two offspring and one cow had three offspring. The relationship between two parent individuals was similar to that between half-sibs. The Ethiopian dataset included 38 cows that each had one offspring, and the Tanzanian dataset included 31 cows and 34 offspring with three of these cows having two offspring.

### Selection of subsets of SNPs

From the 735k SNPs in the Kenyan/Ugandan dataset, subsets of 100, 200, 300, 400, 500, 1000, and 1500 SNPs were chosen based on several selection criteria that are described below, resulting in SNPs located on all chromosomes except for the smaller panels with less than 200 SNPs; the number of SNPs was smaller on short than on long chromosomes. To minimize linkage disequilibrium, SNPs had to be at least one megabase (Mb) pair apart. Some of the methods to select SNPs were carried out within the crossbred population under investigation (e.g. with the highest minor allele frequency (MAF)), which implies that they should, ideally, be repeated when moving to a different population. However, the selected SNP panels were validated in independent crossbred populations to assess the potential for a wider application of our SNP panels. SNP panels were selected based on the criteria described in the following paragraphs.

#### Highest minor allele frequency

Allele frequencies were calculated for the crossbred animals. SNPs were sorted by MAF and subsets were selected based on the highest MAF in the crossbred animals. These subsets are not independent since larger subsets always included SNPs in the smaller subsets. The average distance between SNPs in the smallest and largest panels were 19.4 Mb [standard deviation (SD) = 16.2 Mb] and 1.7 Mb (SD = 0.7 Mb), respectively.

#### Differences in absolute frequency

Allele frequencies were calculated for the ancestral breeds. The weighted average allele frequency across breeds was calculated based on the number of animals in each breed sample. Weighted averages were calculated across the Nelore and N’Dama populations (NelNd) and across all European dairy breeds (EU). The differences in absolute frequencies were determined between Nelore and EU (NelEU), N’Dama and EU (NdEU), and NelNd and EU (NelNdEU). SNPs were sorted according to differences in absolute frequencies and subsets that had the largest differences were selected. As above, these subsets are not independent because larger subsets include all the SNPs in the smaller subsets. The average distance between SNPs per chromosome for the 100-SNP panel was 17.6 Mb (SD = 14.4 Mb), 17.4 Mb (SD = 16.3 Mb), and 15.6 Mb (SD = 14.2 Mb) for NelEU, NdEU, and NelNdEU, respectively. For the 1500-SNP panel, the average distance between SNPs per chromosome was 1.7 Mb (SD = 0.7 Mb), 1.7 Mb (SD 0.7 = Mb), and 1.7 Mb (SD = 0.6 Mb) for NelEU, NdEU, and NelNdEU, respectively.

#### Random selection

We selected 10 random samples for each subset and results were averaged across these random samples. These random panels were not restricted by SNP spacing (i.e. the 1 Mb pair restriction). The average distance between SNPs per chromosome ranged from 20.4 Mb (SD = 16 Mb) for the 100-SNP panel to 1.7 Mb (SD = 1.6 Mb) for the 1500-SNP panel.

#### ISAG panel and 50k-SNP chip

The official International Society for Animal Genetics (ISAG) panel for parentage assignment [24] consists of 100 core SNPs, which are mostly derived from European breeds, plus an additional 100 SNPs from *Bos indicus* animals. We also tested 47,810 SNPs from the Illumina 50k-SNP bovine chip v2 (San Diego, CA, USA).

#### Differential evolution (DE) algorithm

The differential evolution (DE) algorithm is based on Storn and Price [25] and ranks SNPs according to a random key (vector of real values; [26]). This key evolves to a higher rank as the SNP is more suited to solve a particular problem (e.g. estimation of breed proportion or parentage assignment [27, 28]).

In our study, the “all animals” set (including pure and crossbred animals) was split into a training and a test population. The DE algorithm was initiated in the training population with 100 random samples of SNPs (‘parental sets’) for each panel size (i.e. 100, 200, 300, 400, 500, 1000, 1500 SNPs). From these 100 parental sets, two sets were randomly selected to create an ‘offspring set’ consisting of 50% randomly sampled SNPs from each parental set. If this offspring set performed better than the initial 100 parental sets (according to a fitness function), then this offspring set was retained and the worst parental set was discarded. The dairy proportions were estimated internally with a SNP-best linear unbiased prediction (BLUP) approach (see below), whereas the parentage test was based on number of *opH*.

The fitness function used to optimize prediction of dairy breed proportions was the coefficient of determination (r^2^) between the subsets of SNPs and the dairy breed proportions predicted with the 735k SNPs in ADMIXTURE. To optimize parentage assignments, the fitness function was the percentage of correctly assigned parentages according to the reconstructed pedigree. This process was run for 2000 iterations/generations. No spacing restriction between SNPs was applied since the DE algorithm should select best SNPs by default.

The average distance between SNPs per chromosome for the panels to estimate breed proportions ranged from 23.6 Mb (SD = 12.8 Mb) for the 100-SNP panel to 1.6 Mb (SD = 1.6 Mb) for the 1500-SNP panel and for the panels to assign parentage, it ranged from 19.7 Mb (SD = 11.2 Mb) for the 100-SNP panel to 1.7 Mb (SD = 1.6 Mb) for the 1500-SNP panel.

### Accuracy and bias of breed proportion prediction

Total dairy proportion for an animal was the sum of the estimated individual breed proportions for Ayrshire, Guernsey, Jersey, Holstein, and Friesian. Accuracy of the prediction of dairy proportions for all subsets of SNPs was assessed by the coefficient of determination (r^2^) between observed (based on all 735k SNPs) and predicted (based on subsets of SNPs) dairy proportions of the 1933 crossbred animals. The linear bias of breed proportions estimated from subsets of SNPs was assessed as the average deviation or average absolute difference estimated minus the observed (735k SNPs) values.

#### Parentage assignment

The *opH* matrices were calculated for each subset of SNPs in the crossbred population. Subsequently, the separation value (*sv*) was used to quantify and visualize the performance of each SNP subset and was calculated as:

1 $$sv = \hbox{min} \left( {FR} \right){-}\hbox{max} \left( {TR} \right),$$

where *FR* is the number of opposing homozygotes in false parent–offspring relationships according to the reconstructed pedigree information; and *TR* is the number of opposing homozygotes in true parent–offspring relationships [23, 29].

#### Regression and SNP-BLUP

Prediction of breed proportions was also made with a regression model and a SNP-BLUP approach to test the ability of the SNP panels to perform outside ADMIXTURE.

The regression method was based on Kuehn et al. [30] and described in Dodds et al. [31] for prediction of breed proportions:

2 $${\mathbf{y}} = {\mathbf{X}}{\hat{\mathbf{b}}} + {\mathbf{e}},$$

where ${\mathbf{y}}$ are the proportions of the designated allele in the genotypes for each SNP of each animal (encoded as allele counts 0, 0.5, 1); ${\mathbf{X}}$ is a matrix of allele frequencies in each reference breed (ADMIXTURE P-file output); ${\hat{\mathbf{b}}}$ are the breed proportions of each animal for each reference breed (to be estimated); and ${\mathbf{e}}$ are the residual errors. Coefficients of determination were calculated between predictions of breed proportions in the ADMIXTURE analysis (735k SNPs) and predictions of the regression method. In addition, the ADMIXTURE P-file was replaced by observed allele frequencies in the ancestral populations.

The SNP-BLUP approach required the replacement of missing genotypes (NA) with the average allele count across all animals. Only SNPs with a call rate higher than 95% were used in this analysis to limit potential bias due to SNPs with only a few recorded genotypes. SNP-BLUP was performed as follows:

3 $$\left[ {{\mathbf{ZZ}}^{{\prime }} + {\mathbf{I}}\uplambda} \right] *{\hat{\mathbf{g}}} = \left[ {{\mathbf{Zy}}} \right],$$

where ${\hat{\mathbf{g}}}$ is the effect of the SNPs to be estimated; ${\mathbf{y}}$ is a vector of dairy proportions (ADMIXTURE output for 735k SNPs) scaled with a mean = 0 and SD = 1; ${\mathbf{Z}}$ is a design matrix allocating SNP genotypes (multiplied by their allele frequencies) to records; ${\mathbf{I}}$ is an identity matrix and $\uplambda$ defines the contribution of genomic relationships. $\uplambda$ was set to $\uplambda = \left( {1 - {\text{h}}^{2} } \right)/({\text{h}}^{2} /{\text{d}})$ with the heritability assumed to be ${\text{h}}^{2}$ = 0.99 and ${\text{d}}$ representing the average heterozygosity of the panel.

SNP effects (${\hat{\mathbf{g}}}$) were subsequently multiplied by ${\mathbf{Z}}$ to obtain estimates of dairy proportions (i.e. GEBV) for each panel. The estimated dairy proportions had to be rescaled (reversing the scaling of ${\mathbf{y}}$) to be correctly interpreted. This approach was also used within the DE algorithm.

#### Validation

When SNPs are selected based on information that is independent of the test dataset, there is no ascertainment bias. Selection of SNPs based on MAF in the crossbred population is subject to trivial ascertainment bias due to binomial sampling variance of allele frequencies (approximately ± 0.01).

The linear regression and SNP-BLUP estimates of breed proportions are subject to ascertainment bias and thus require validation. Validation was achieved by using the SNP effects that were estimated in the Kenyan/Ugandan dataset to predict dairy proportions in the Ethiopian and Tanzanian dataset and vice versa. To determine whether population structure or random sampling caused bias in the estimates, we applied the SNP-BLUP approach to predict breed proportions for 50% of the animals in the Kenyan/Ugandan dataset by randomly selecting 50% of the animals in each breed (training dataset). Then, cross-validation of the estimates was performed on the other half of the Kenyan/Ugandan population as well as on the Ethiopian and Tanzanian crossbred animals (test datasets).

We further validated our sets of SNPs in independent crossbred populations. The subsets of SNPs that were selected from the Kenyan/Ugandan dataset were used to predict breed proportions and parentage assignment in the Ethiopian and Tanzanian datasets. The coefficient of determination and the absolute linear bias between the full dataset and the subsets within the Ethiopian and Tanzanian datasets were used to determine the performance of each subset of SNPs to accurately assign dairy proportions, and the *sv* was used for parentage assignment.

## Results and discussion

### Description of data

After merging the quality-controlled datasets for each breed, 4.8, 5.1, and 5.8% of genotypes were missing in the entire Kenyan/Ugandan, Ethiopian, and Tanzanian datasets, respectively. Within the crossbred animals, 4.9, 5.5, and 4.8% of genotypes were missing in the Kenyan/Ugandan, Ethiopian, and Tanzanian datasets, respectively. There was no general pattern of where the missing genotypes occurred along the genome.

The average inbreeding coefficient (*F*_IS_) did not show any substantial average inbreeding in any of the breeds; however, the SD was very large (Table 1). The genomic relationship matrix (GRM) showed that the crossbred animals were mostly unrelated with no detectable inbreeding (Table 1). The assumed ancestral breeds included related individuals within the range of half-sib relations. Exceptions were the N’Dama and Nelore populations in which individuals appeared to be highly related and inbred, with Nelore showing an average diagonal element of 1.82 (Table 1). The high values of the GRM for the Nelore population can be explained by ascertainment bias [32] combined with how the GRM is calculated. Nelore is a pure *Bos indicus* breed and N’Dama represents a unique African *Bos taurus* breed. The largest proportion of SNPs on the 735k-Illumina chip was chosen based on high information content (high MAF) within non-African *Bos taurus* populations.

**Table 1:** Average diagonal and off-diagonal elements of the GRM [18] and inbreeding coefficient (*F*_*IS*_) ± SD (SE) in cattle

	Diag	Off-Diag	*F*_IS_
Ayrshire	1.11 ± 0.044 (0.01)	0.34 ± 0.089 (0.02)	−0.024 ± 0.206 (0.05)
Friesian	1.01 ± 0.025 (0.005)	0.18 ± 0.05 (0.01)	−0.005 ± 0.191 (0.04)
Guernsey	1.16 ± 0.036 (0.008)	0.37 ± 0.089 (0.02)	0.018 ± 0.218 (0.05)
Holstein	1.11 ± 0.036 (0.008)	0.29 ± 0.089 (0.02)	−0.021 ± 0.210 (0.05)
Jersey	1.21 ± 0.040 (0.009)	0.48 ± 0.134 (0.03)	−0.0003 ± 0.218 (0.05)
N’Dama	1.28 ± 0.022 (0.005)	0.65 ± 0.027 (0.009)	0.013 ± 0.215 (0.05)
Nelore	1.82 ± 0.036 (0.008)	1.22 ± 0.045 (0.01)	0.003 ± 0.209 (0.05)
XBred^a^	0.98 ± 0.044 (0.001)	0.0004 ± 0.044 (0.001)	0.024 ± 0.037 (0.0008)
E XBred^a^	0.95 ± 0.070 (0.003)	0.01 ± 0.070 (0.003)	0.015 ± 0.050 (0.002)
T XBred^a^	0.954 ± 0.003 (0.0001)	0.004 ± 0.002 (0.0001)	0.032 ± 0.06 (0.003)

^a^XBred, Kenya/Uganda; E XBred, Ethiopia; T XBred, Tanzania

**Fig. 1:**
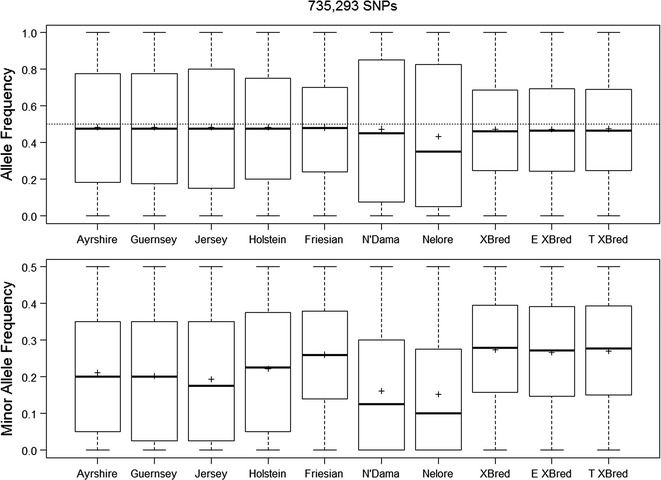
Allele frequencies and minor allele frequencies for seven ancestral and three crossbred (XBred) cattle breeds. *Bold horizontal lines* indicate the median and *plus symbol* indicates the mean; the *box* for each population indicates the interquartile range, and the outermost *bars* for each population indicate the most extreme observations. XBred= Kenya/Uganda; E XBred= Ethiopia; T XBred= Tanzania

Figure 1 shows MAF and absolute allele frequencies for the various populations in our analyses and clearly illustrates the bias that is due to the criteria applied for selecting SNPs in the assay. The method of constructing the GRM across multiple breeds [18] centers the matrix by using most of the animals in the dataset. Thus, the level of inbreeding appears to be high in the N’Dama and Nelore populations, which represent a small number of animals and they have MAF that clearly differ from those of other groups. When the GRM was constructed by using only the Nelore animals (n = 20), the average of the diagonal elements was equal to 0.975, which is consistent with the diagonal elements of the GRM for Nelore reported by Zavarez et al. [33].

The allele frequencies for the three crossbred populations showed a narrower inter-quartile range compared to the assumed ancestral populations (Fig. 1). Compared to the other breeds, more than twice the number of SNPs were not in Hardy–Weinberg equilibrium (the null-hypothesis was rejected) in the crossbred populations (151,486 SNPs for the Kenyan/Ugandan, 58,493 for the Ethiopian, and 91,460 for the Tanzanian datasets), which is likely due to a proportion of the crossbred animals originating from the first generation progeny of crosses with pure dairy or indigenous breeds.

**Fig. 2:**
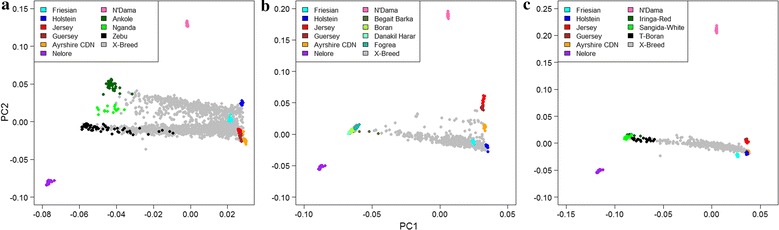
Principal components for exotic and indigenous cattle populations **a** Kenya/Uganda, **b** Ethiopia, and **c** Tanzania. PC1 separates European from African breeds. PC2 separates Nelore and N’Dama breeds and African and European breeds

A principal component (PC) analysis based on the GRM for the combined Kenyan and Ugandan dataset separated the European dairy breeds from the Nelore breed and then from the African pure breeds and the first PC explained 86.91% of the genetic variation. The second PC clearly separated the Nelore and N’Dama breeds from the European breeds and crossbreds, with the East African indigenous breeds being intermediate; it explained 1.75% of the genetic variation (Fig. 2a). The second PC also separated Kenyan and Ugandan crossbred animals, which spread between their respective indigenous breeds (Ankole and Nganda in Uganda and Zebu in Kenya) and European ancestral breeds. Although the indigenous samples were collected from animals that phenotypically appeared as pure indigenous, they clearly included animals that were admixed with European *Bos taurus* genes. When considering only the clusters of apparently pure indigenous animals, the variation between these three indigenous breeds was substantially larger in both dimensions (PC1 and PC2) than the difference between the European *Bos taurus* dairy breeds.

The first two PC in the Ethiopian dataset explained 90.92 and 1.71% of the variation, respectively (Fig. 2b), and in the Tanzanian dataset, they explained 85.19 and 3.23% of the variation, respectively (Fig. 2c). Most of the Ethiopian crossbreds aligned with their respective indigenous breeds and with the Friesian and Holstein breeds; however, some animals were positioned between an unknown indigenous population and the Ayrshire population. The Tanzanian crossbred animals were positioned between their indigenous breeds and the Holstein and Ayrshire breeds. However, when all the data from DGEA1 and 2 were analyzed simultaneously and the results were plotted to show the third PC, the Tanzanian crossbred animals were closer to the Friesian breed (see Additional file 1: Figure S1). Similar to the Ethiopian crossbreds, some Tanzanian crossbreds seemed to align with an unknown indigenous breed (Fig. 2c). The three-dimension PCA plot for the analysis that included indigenous breeds from all countries, showed that the Ethiopian and Tanzanian crossbred animals that were not aligned to a local indigenous breed, aligned with an unknown breed(s) between the East African Zebu and the Nganda breed (see Additional file 1: Figure S1). Crossbred animals from Tanzania that did not align with a local breed in the analysis were sampled from the Southern Highlands, whereas those from Ethiopia came from various locations across the country.

### Description of the SNP panels

**Fig. 3:**
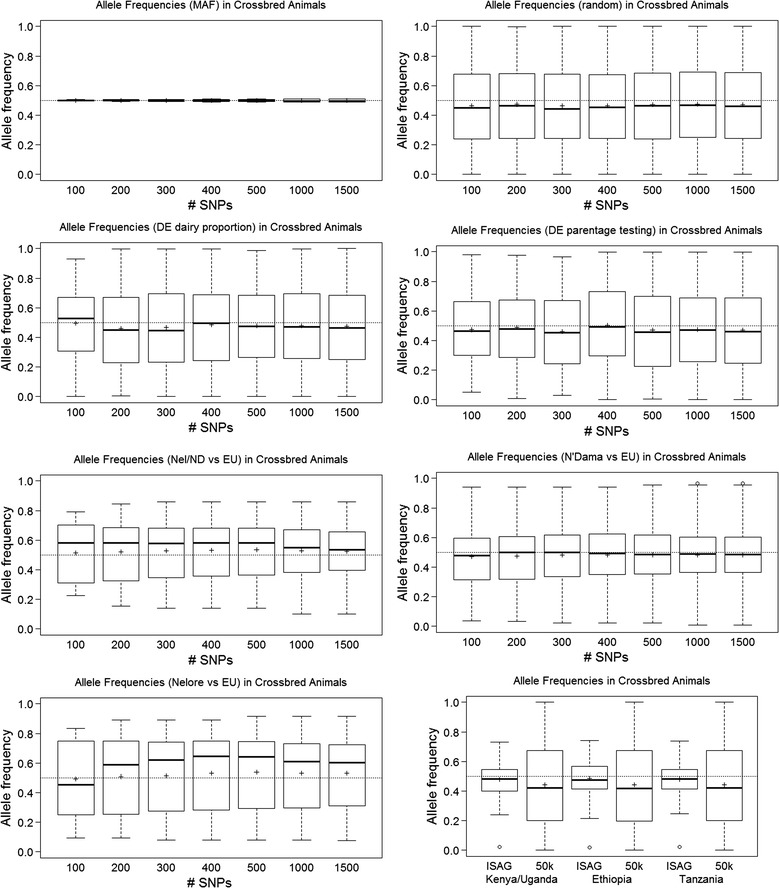
Allele frequencies for SNP panels in a crossbred cattle population (Kenya/Uganda). *Bold horizontal lines* indicate the median and *plus symbol* indicates the mean; MAF, highest minor allele frequencies; DE, differential evolution algorithm; Nel/ND versus EU, Nelore versus EU; N’Dama versus EU, highest absolute allele frequency difference

As expected given the sampling procedure applied, panels of SNPs that were selected on their highest MAF showed almost no variation in allele frequencies for the Kenya/Uganda crossbred animals with median and mean allele frequencies at 0.5. The panel that showed the next to lowest variation was the combined NdEU panel for which the interquartile range was between 0.35 and 0.6 (Fig. 3).

All other selection methods resulted in relatively large interquartile ranges. Deviation in mean and median allele frequency was largest for the NelEU panels (Fig. 3). Examination of these allele frequencies within the Nelore and EU breeds revealed that the shift in frequencies was very similar but in opposite directions for the European breeds versus the Nelore breed for all SNP panels (see Additional file 2: Figure S2). The observed frequency for the NelEU SNP panel in the crossbred animals (Fig. 3) most likely reflects that the average crossbred animal in this population was 69.7% (SD = 21.1%) European *Bos taurus*. A similar but less extreme effect was found for the NdEU SNP panel (see Additional file 2: Figure S2).

The ISAG SNP panel showed a narrow inter-quartile range with a mean and median at 0.5, and the inter-quartile range of the 50k-SNP panel was similar to that of the full 735k-SNP panel although the mean and median frequencies deviated more from 0.5 (Fig. 3).

The distributions of allele frequencies in the Ethiopian and Tanzanian crossbred animals were similar to that in the Kenya/Uganda crossbred animals, but with a wider range of frequencies for all SNP panels (see Additional file 3: Figure S3, Additional file 4: Figure S4).

### Estimation of breed proportions

**Fig. 4:**
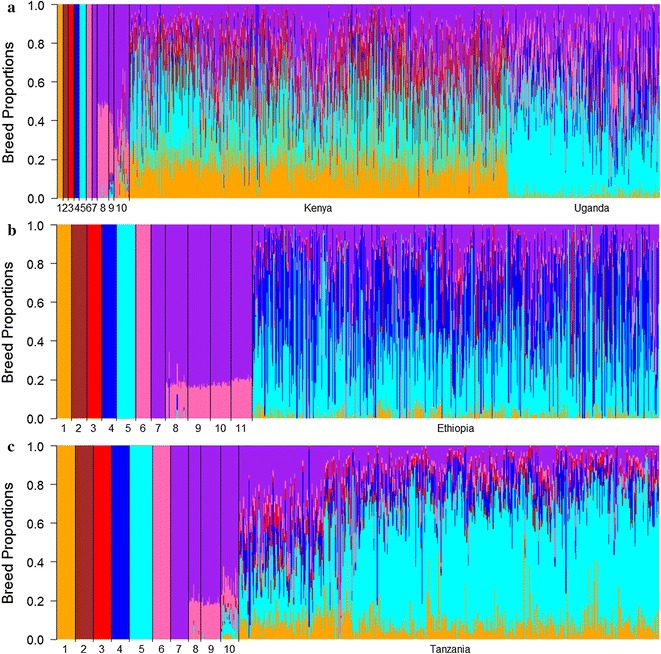
Breed proportions of crossbred dairy cattle **a** Kenya/Uganda, **b** Ethiopia, and **c** Tanzania. Supervised ADMIXTURE analysis with seven fixed ancestral breeds: **a** *1* Ayrshire, *2* Guernsey, *3* Jersey, *4* Holstein, *5* Friesian, *6* N’Dama, *7* Nelore, *8* Ankole, *9* Nganda, *10* Zebu. **b** *1* Ayrshire, *2* Guernsey, *3* Jersey, *4* Holstein, *5* Friesian, *6* N’Dama, *7* Nelore, *8* Begait Barka, *9* Danakil Harar, *10* Ethiopian Boran, *11* Fogera. **c** *1* Ayrshire, *2* Guernsey, *3* Jersey, *4* Holstein, *5* Friesian, *6* N’Dama, *7* Nelore, *8* Iringa Red, *9* Singida White, *10* Tanzanian Boran

Proportions of dairy breed in the crossbred animals were on average equal to 0.70 (SD = 0.21), 0.78 (SD = 0.20), and 0.78 (SD = 0.18) for the Kenyan/Ugandan, Ethiopian, and Tanzanian datasets, respectively. This proportion was highest for Ayrshire in Kenyan crossbred animals and for Friesian in Ugandan crossbred animals, which was consistent with the PCA results (Fig. 4). Based on a study of smallholder cattle that were sampled from mostly peri-urban areas in Kenya, Gorbach et al. [12] reported that the crossbred cattle had very high dairy breed proportions, which reflected the fact that their samples originated from a much smaller, more intensive and older dairy production area than in our study. They found that the main dairy breeds present in the crossbred individuals were Holstein and Jersey/Guernsey, the latter two being indistinguishable. However, their analysis did not include Ayrshire as a reference breed and Weerasinghe [15] showed that when Ayrshire was excluded from the ADMIXTURE analysis, most of the Ayrshire signal appeared as Guernsey or Jersey.

In Ethiopian crossbred animals, proportions of dairy breeds were highest for Holstein and Friesian (Fig. 4), which was consistent with the PCA analyses and the documented history of the country’s specific cattle imports [15]. In Tanzanian crossbred animals, the Friesian breed proportion was highest, which differed from the results of the PCA in which they aligned more closely with the Holstein and Ayrshire breeds (Fig. 4). Dairy breed proportions were highest in crossbred animals from the Southern Highland sampling site (0.84, SD = 0.12) compared to the other Tanzanian crossbreds, which was consistent with the PCA.

We selected three SNP panels (NelEU, NdEU, and NelNdEU) based on the largest differences in allele frequency between ancestral breeds. Our hypothesis was that SNPs that display the largest difference in allele frequency between the indigenous ancestral breeds and the dairy breeds will provide the most accurate estimates of total dairy breed proportion. Total dairy breed proportion was defined as the sum of breed proportions across Ayrshire, Guernsey, Jersey, Holstein, and Friesian breeds. Panels of SNPs that were selected by applying other methods were included to investigate the factors that determine accuracy of prediction and whether it was possible to develop SNP panels that could estimate both breed proportion and parentage assignment.

**Fig. 5:**
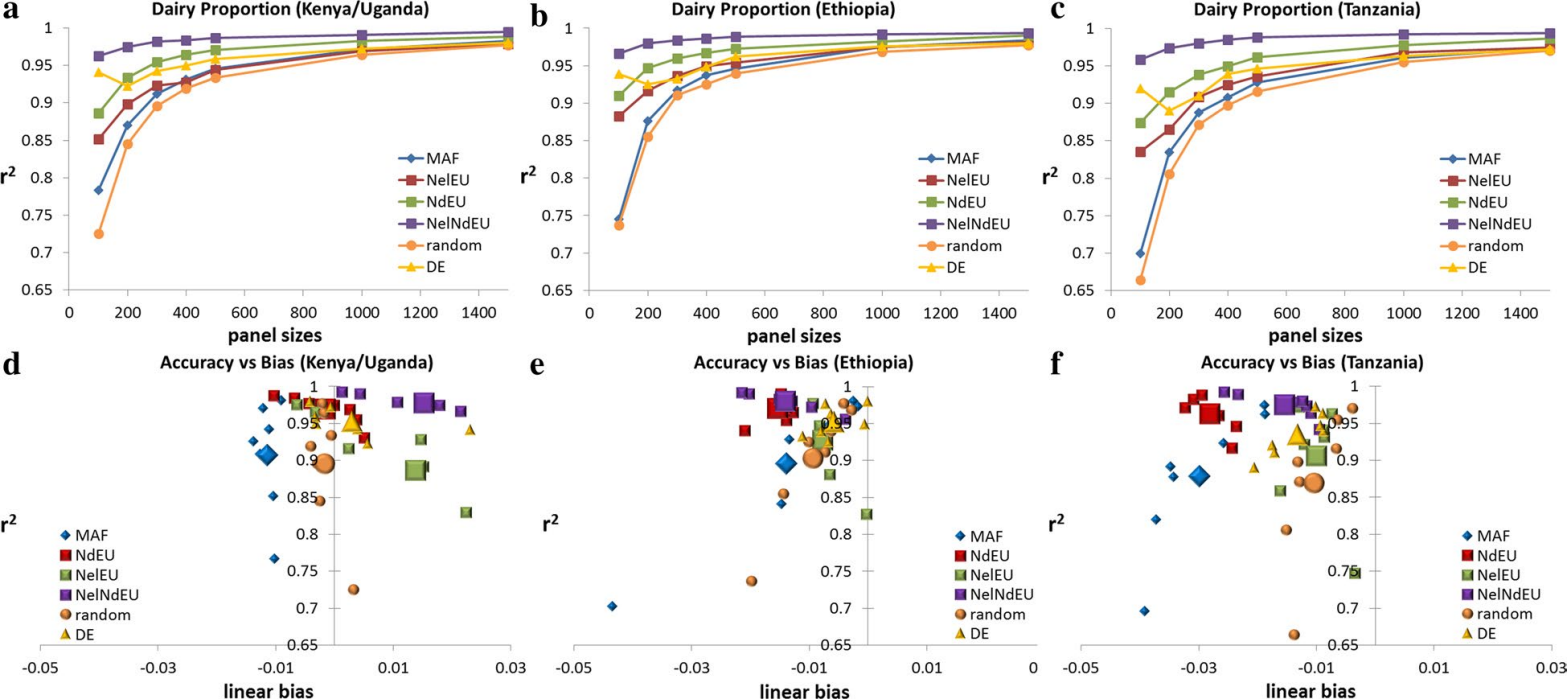
Accuracy (r^2^) of dairy proportion estimates (**a**–**c**) and accuracy versus bias (**d**–**f**) for different panel sizes. **d**–**f** large symbols show average linear bias across all panel sizes. Standard errors of accuracy ranged on average from 0.008 for 100 SNPs to 0.003 for 1500 SNPs (Kenya/Uganda), 0.015 to 0.005 (Ethiopia), and 0.02 to 0.008 (Tanzania)

The various panels used in this study predicted dairy breed proportions in the Kenyan/Ugandan crossbreds with an r^2^ of 0.725 to 0.963 (SE = 0.004–0.012) for the smallest subsets of 100 SNPs, and 0.977–0.994 (SE = 0.002–0.003) for the largest subsets of 1500 SNPs (Fig. 5a). As hypothesized, the NelNdEU SNP panel achieved the best results for all panel sizes, with an r^2^ of 0.974 (SE = 0.004) with just 200 SNPs. The next best panel was the NdEU for all panel sizes except 100 SNPs, for which the DE algorithm performed slightly better (Fig. 5a). Surprisingly, the NelEU SNP panel performed worse compared to the other panels selected for largest differences in allele frequency, with an r^2^ of 0.852 (SE = 0.009) and 0.898 (SE = 0.007) for 100 and 200 SNPs, respectively, because as shown by Table 2 NelEU SNPs are efficient for distinguishing *Bos taurus* from *Bos indicus* but not for separating African from European *Bos taurus*.

**Table 2:** Accuracies (r^2^) of individual breed proportions in crossbred dairy cattle (Kenya/Uganda) for 200 SNPs selected by different methods

	Dairy	Ayrshire	Friesian	Guernsey	Holstein	Jersey	N’Dama	Nelore	Mean ± SD^a^
MAF	0.870	0.486	0.312	0.375	0.483	0.165	0.397	0.874	0.442 ± 0.22
NelEU	0.898	0.120	0.140	0.056	0.198	0.034	0.484	0.944	0.282 ± 0.33
NdEU	0.934	0.373	0.263	0.265	0.437	0.141	0.538	0.867	0.412 ± 0.24
NelNdEU	0.974	0.075	0.132	0.013	0.007	0.004	0.511	0.190	0.133 ± 0.18
Random	0.845	0.405	0.243	0.276	0.414	0.133	0.373	0.874	0.388 ± 0.24
DE	0.922	0.421	0.049	0.281	0.402	0.141	0.427	0.925	0.378 ± 0.28
ISAG	0.831	0.452	0.197	0.270	0.306	0.127	0.482	0.759	0.378 ± 0.21
Mean ± SD	0.896 ± 0.05	0.333 ± 0.17	0.197 ± 0.09	0.219 ± 0.13	0.321 ± 0.17	0.107 ± 0.06	0.459 ± 0.06	0.776 ± 0.27	

Standard errors of breed-wise accuracies ranged from 0.017 to 0.02

^a^Excluding dairy

The performance of the panels selected with the DE algorithm did not improve much as the number of SNPs increased, and hence were outperformed by the NelNdEU and NdEU panels for more than 100 SNPs. The DE algorithm was designed to optimize a panel for the prediction of the 735k ADMIXTURE estimates of dairy proportions. However, SNP-BLUP was used to estimate dairy proportions, rather than ADMIXTURE as for all other panels. In addition, when we predicted dairy proportions by using a SNP-BLUP approach independently of the DE algorithm (see next section), the DE-based panels continued to perform less well than the NelNdEU and NdEU panels, which indicated that the DE algorithm failed to find the optimal solution with the number of iterations performed. Esquivelzeta-Rabell et al. [28] used the DE algorithm to predict Korean Hanwoo proportions in a Chinese Yeonbyun population and reported r^2^ of 0.69 and 0.88 for 100 and 1000 SNPs, respectively. These coefficients of determination are lower than those obtained by using the same number of SNPs and the DE algorithm but the genetic differences between Yeonbyun and Hanwoo are much smaller than those between indigenous and European dairy breeds in our study.

Figure 5d shows the relationship between bias and accuracy for all methods of SNP selection and size of SNP panels in each of the three populations. The average absolute linear bias was smallest for the NelNdEU panel (0.026, SD = 0.009) followed by the NdEU (0.035, SD = 0.01), and the DE panel (0.036, SD = 0.009).

When regressing 735k SNP predictions on either the 200 or 400 SNP predictions (see Additional file 5: Figure S5, Additional file 6: Figure S6) for the best panel (NelNd:EU), the slope was greater than 1.0, with the highest bias obtained for low dairy breed proportions. ADMIXTURE forces estimates of breed proportions to be between 0 and 1, which might lead to an inherent bias at either end of the range of breed proportion estimates. To assess whether the linear bias stemmed intrinsically from this constraint on the ADMIXTURE estimates and whether a correction factor could be introduced, for the 200 and 400 NelNdEU SNP panels, we truncated the range of dairy proportion estimates from the 735k-SNP panel to between 0.1 and 0.9 or between 0.2 and 0.8. Absolute biases were not much affected by truncation of the data; they increased slightly at high dairy breed proportions and decreased slightly at low proportions (see Additional file 5: Figure S5, Additional file 6: Figure S6).

The estimates of dairy proportions were slightly more accurate and less biased for the Kenyan crossbred animals (r^2^ = 0.972, SE = 0.005; average absolute bias = 0.028 SD = 0.022) than for the Ugandan crossbred animals (r^2^ = 0.963, SE = 0.008; average absolute bias = 0.04, SD = 0.03). Kenyan crossbred animals are the result of crosses between European dairy breeds and Zebu whereas Ugandan crossbreds are crosses between European dairy breeds and Ankole and Nganda, which have much higher proportions of African *Bos taurus* ancestry than Zebu. This suggests that the bias observed for the crossbreds in these two countries is predominantly due to a tendency to over-predict the African *Bos taurus* proportion and under-predict the European *Bos taurus* proportion.

Validation of SNP panels in the Ethiopian and Tanzanian crossbred animals resulted in a similar ranking with the NelNdEU panel performing best for all panel sizes, and resulting in r^2^ of 0.966, 0.980, and 0.993 in Ethiopian crossbreds, and 0.958, 0.974, and 0.994 in Tanzanian crossbreds for 100, 200, and 1500 SNPs, respectively (Fig. 5b, c). The worse performance was observed for the random panel closely followed by the MAF panel with r^2^ of 0.745 and 0.699 (SE = 0.022 and 0.026) in Ethiopian and Tanzanian crossbreds for 100 SNPs, respectively, compared to an r^2^ of 0.783 (SE = 0.012) in the Kenyan/Ugandan population. Average absolute bias was smallest for the NelNdEU panel in both datasets (0.024, SD = 0.003 for the Ethiopian dataset and 0.026, SD = 0.002 for the Tanzanian dataset), followed by the NdEU panel (0.033, SD = 0.009 for the Ethiopian dataset and 0.041, SD = 0.009 for the Tanzanian dataset). In both countries, panels under-predicted dairy proportions except for some NelEU panel sizes in Tanzania (Fig. 5e, f).

The full ISAG panel of 200 SNPs predicted dairy proportions with r^2^ of 0.831 (SE = 0.009), 0.830 (SE = 0.018), and 0.768 (SE = 0.022) in the Kenyan/Ugandan, Ethiopian, and Tanzanian datasets. Average absolute bias of the ISAG panel was among the highest values for the 200-SNP panels, i.e. 0.069, 0.067, and 0.076 in the Kenyan/Ugandan, Ethiopian, and Tanzanian datasets, respectively. This poor performance is not unexpected since the ISAG panel was selected for parentage assignment, predominantly in *Bos taurus* populations. SNPs on the 50k-SNP v2 Illumina chip predicted dairy proportions with r^2^ of 0.9987 (SE = 0.0008), 0.9989 (SE = 0.001), and 0.9985 (SE = 0.002) in the Kenyan/Ugandan, Ethiopian, and Tanzanian datasets, respectively. Absolute bias of predicted dairy proportions was equal to 0.006, 0.005, and 0.009 for the Kenyan/Ugandan, Ethiopian, and Tanzanian datasets, respectively.

When predicting the proportions of each of the seven ancestral breeds based on the various 200-SNP panels (Table 2), average accuracies across ancestral breeds were highest for selection of SNPs based on MAF (r^2^ = 0.442, SE = 0.017) followed by the NdEU SNP selection (r^2^ = 0.412, SE = 0.017). The panels based on maximizing indigenous versus dairy allele frequencies including the Nelore breed (NelNdEU, NelEU) gave very poor predictions of individual dairy breed proportions. This is due to the selection method that preferentially selects alleles at extreme frequencies between *Bos taurus* and *Bos indicus* breeds and hence results in low variance between dairy breeds.

With subsets of 200 SNPs, individual breed proportions were on average best predicted for the Nelore breed (r^2^ = 0.776, SE = 0.009) followed by the N’Dama breed (r^2^ = 0.459, SE = 0.017). Jersey breed proportions were poorly predicted with on average an r^2^ of 0.107 (SE = 0.021, Table 2). The accuracy of individual breed proportion predictions is strongly influenced by two factors: (1) breeds that exhibit little variation in breed proportions in the crossbred animals (such as Jersey) will have their proportions predicted with lower r^2^ since the residual errors account for a higher proportion of the total variation accuracy; and (2) breeds that are most genetically distant from the others (such as the Nelore breed) are more likely to display allele frequencies that differ from those of other breeds with most methods used to select SNP panels.

The NelEU panel performed well for the prediction of Nelore proportion, with an r^2^ of 0.944 (SE = 0.005), but gave poorer predictions of total dairy proportion (r^2^ = 0.484, SE = 0.016) because of its poor prediction of N’Dama (African *Bos taurus*) versus European *Bos taurus* proportions. Although this panel was not as good at predicting total dairy proportion in these African crossbred populations, it should perform better in populations in which the indigenous population is pure *Bos indicus*, as is the case in much of India.

Separating the crossbred animals according to their country of origin (Kenya vs. Uganda) improved the prediction of Nelore proportion in the Kenyan crossbred animals and of breed proportion for Holstein, Jersey, and N’Dama in the Ugandan crossbred animals with most panels. Ayrshire and Guernsey predictions were less accurate with most panels in both Kenyan and Ugandan crossbred animals.

### Regression and SNP-BLUP

**Fig. 6:**
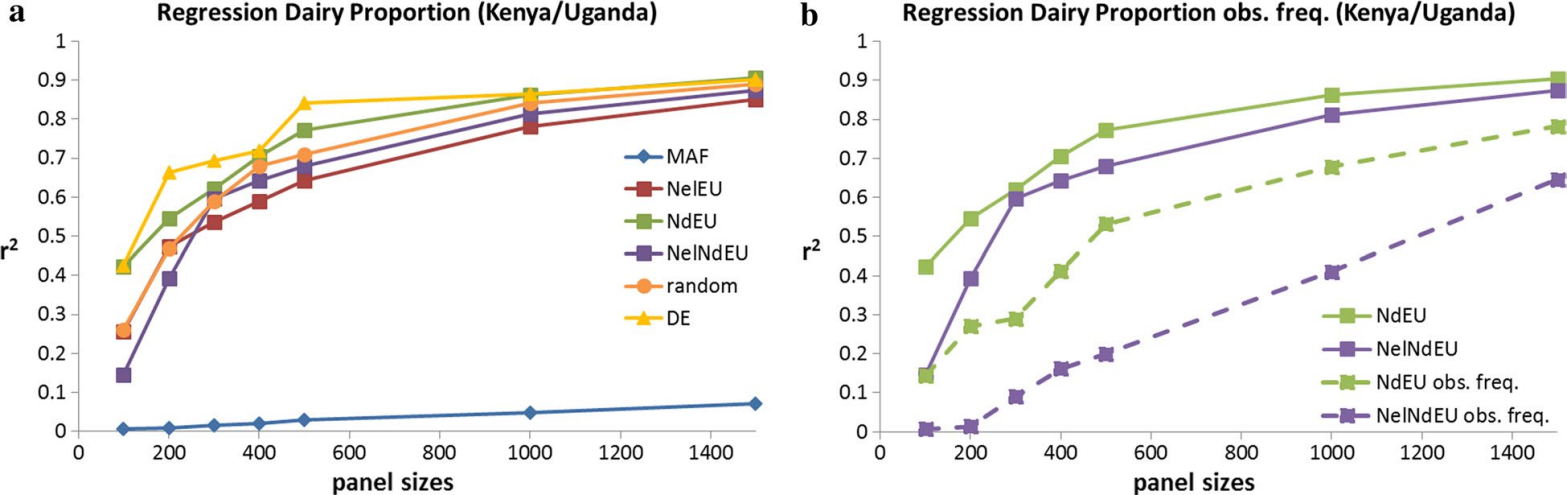
Accuracy (r^2^) of dairy proportion estimates for different panel sizes using a regression approach. **a** ADMIXTURE allele frequencies (P-file). **b** Observed allele frequencies. Standard errors of accuracy ranged on average from 0.02 for 100 SNPs to 0.01 for 1500 SNPs

The regression method using all 735k SNPs predicted dairy proportions from a 735 k ADMIXTURE analysis with an r^2^ of 0.9914 (SE = 0.002) and an absolute bias of 0.014 (SD = 0.018). However, the selected SNP panels gave poor predictions when using the regression method (Fig. 6a). Using 50k SNPs to predict breed proportions in sheep, Dodds et al. [31] reported accuracies of r^2^ = 0.941. Frkonja et al. [34] used different prediction methods and compared the results to pedigree-based admixture estimates. All methods resulted in fairly low r^2^ values (0.872–0.953) with 40,000 SNPs, with a partial least square regression approach performing best. Frkonja et al. [34] also reported no substantial loss in accuracy when the number of SNPs dropped to 4000 (r^2^ = 0.949), but observed a significant loss in accuracy when it dropped to 400 (r^2^ = 0.912). Our results show that the regression approach performs much worse than ADMIXTURE even with 1500 SNPs (Fig. 6a).

We tested the NelNdEU and NdEU panels to determine whether the use of true allele frequencies improved prediction of breed proportions compared to that of ADMIXTURE estimates, and found that prediction accuracy (r^2^) decreased substantially (Fig. 6b).

**Fig. 7:**
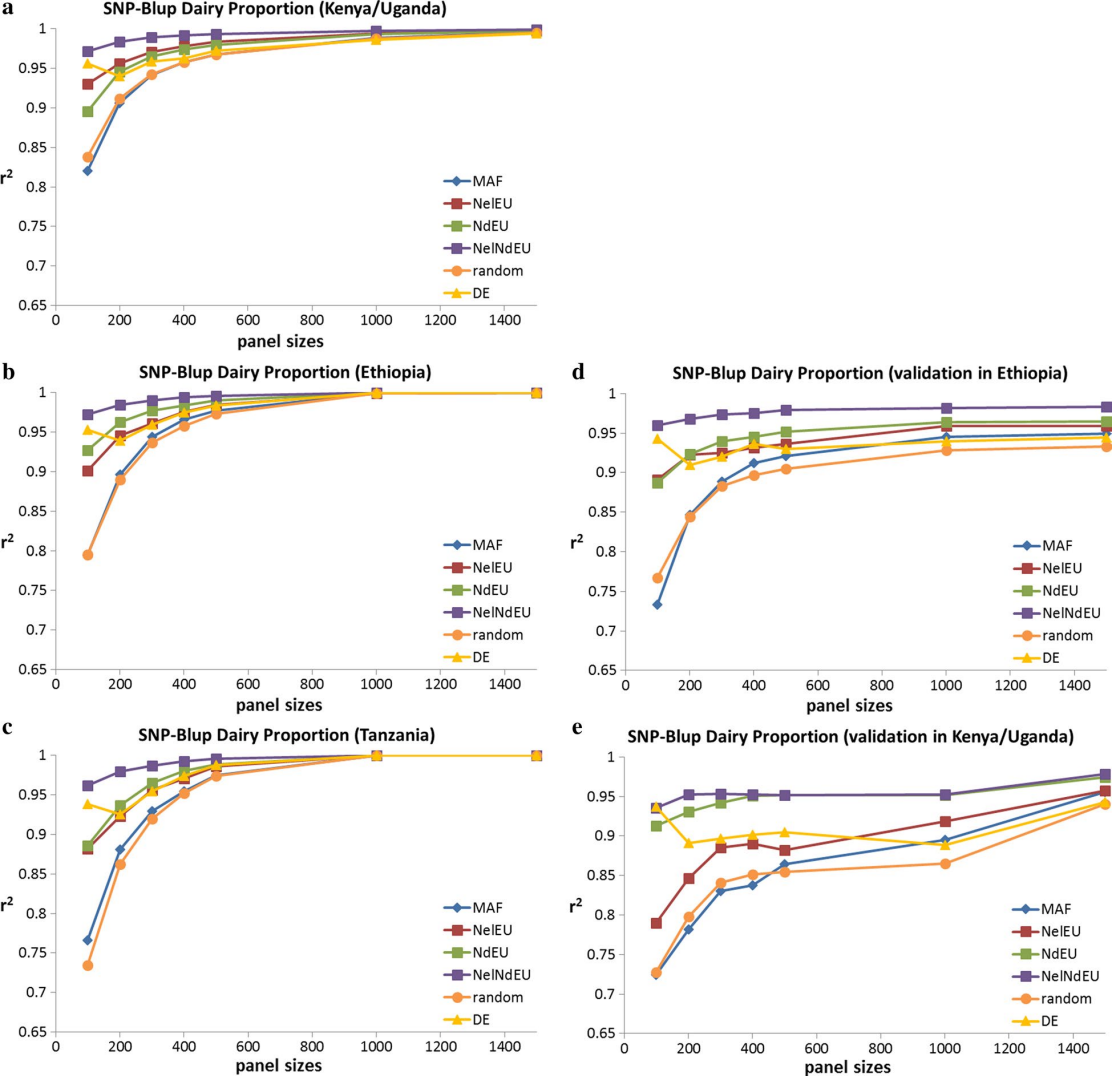
Accuracy (r^2^) and validation of dairy proportion estimates using a SNP-BLUP approach. **a**–**c** Discovery of SNP effects in three independent populations. **d**, **e** Validation of SNP effects estimated in the Kenyan/Ugandan dataset for two independent populations. Standard errors of accuracy ranged on average from 0.006 for 100 SNPs to 0.001 for 1500 SNPs in the Kenyan/Ugandan dataset

When we applied the SNP-BLUP approach with each of the SNP panels and using all the data from each of the three populations, estimates of dairy proportions were more accurate than those obtained by ADMIXTURE except for the NdEU panel, and even much more than those obtained by the regression approach, as shown by the comparison of Fig. 7a–c with Fig. 5a–c. For example, in the Kenyan/Ugandan dataset (Fig. 7a), the NelNdEU panel achieved on average 0.008 higher r^2^ values and the MAF panel 0.026 higher r^2^ values with SNP-BLUP estimates compared to ADMIXTURE estimates. Estimates obtained by using ADMIXTURE are unbiased, because estimates for each SNP panel are obtained independently of the estimates using the 735k SNPs, against which they are tested. In contrast, the accuracies of SNP-BLUP estimates are subject to ascertainment bias that leads to overestimated accuracies. When the prediction equations obtained with SNP-BLUP for the Kenyan/Ugandan dataset were validated by applying them to the Ethiopian and Tanzanian datasets (Fig. 7d, e), accuracies of all panels were substantially lower and always higher with ADMIXTURE. A cross-validation within the Kenyan/Ugandan dataset resulted in similarly lower accuracies, which indicates that dairy proportions are generally overestimated due to ascertainment bias with the SNP-BLUP approach.

To assess whether this ascertainment bias results from random sampling or population structure, we used the NelNdEU panels and split the Kenyan/Ugandan dataset randomly into two equally-sized subsets. The numbers of animals from Kenya and Uganda were relatively evenly distributed between the two subsets. Accuracies of the predictions of dairy proportions obtained with SNP-BLUP on the first half of the dataset were very similar to those obtained using the full dataset. When SNP-BLUP equations were validated on the second half of the dataset, accuracies dropped and biases increased substantially. Using the effects of SNPs that were predicted with the first half of the Kenyan/Ugandan dataset to predict dairy proportions in the second half of the dataset (within-population validation) led to decreased accuracy [average reduction in r^2^ = −0.012, SD = 0.004; (see Additional file 7: Figure S7a)] and increased bias. When these prediction equations were used on the Ethiopian and Tanzanian datasets, accuracies were lower (average r^2^ = 0.417, SE = 0.02 for the Ethiopian dataset and average r^2^ = 0.485, SE = 0.019 for the Tanzanian dataset) (see Additional file 7: Figure S7b). The reduction in accuracy in the within-population validation reflects ascertainment bias due to random sampling. However, reduction in accuracy was even larger in the cross-population validation, which indicates that population structure has a stronger impact on ascertainment bias. Since the SNP-BLUP approach incorporates the allele frequencies of the fitted dataset, predictions of dairy proportions will be less accurate and more biased if the validation dataset includes populations with different allele frequencies (population structure). On average, absolute differences in allele frequency were equal to 0.048 (SD = 0.039) and 0.031 (SD = 0.025) between Kenyan/Ugandan and Ethiopian, and Kenyan/Ugandan and Tanzanian datasets, respectively.

The poor performance of the estimates obtained with the panels that were optimized by using the DE algorithm indicates that either it did not properly search the parameter space to find the optimum panel, or that the number of iterations was insufficient to evolve to the optimum. Figure S8 (see Additional file 8: Figure S8) shows that accuracy continued to increase slowly after 10,000 iterations of the DE algorithm. The curve was too flat to make any prediction about what the asymptotic accuracy might be if a much larger number of iterations was run. However, given the substantial drops in accuracy seen in the validation datasets, there is no reason to believe that the DE algorithm would eventually produce more accurate estimates after validation than ADMIXTURE estimates.

### Parentage assignment

**Fig. 8:**
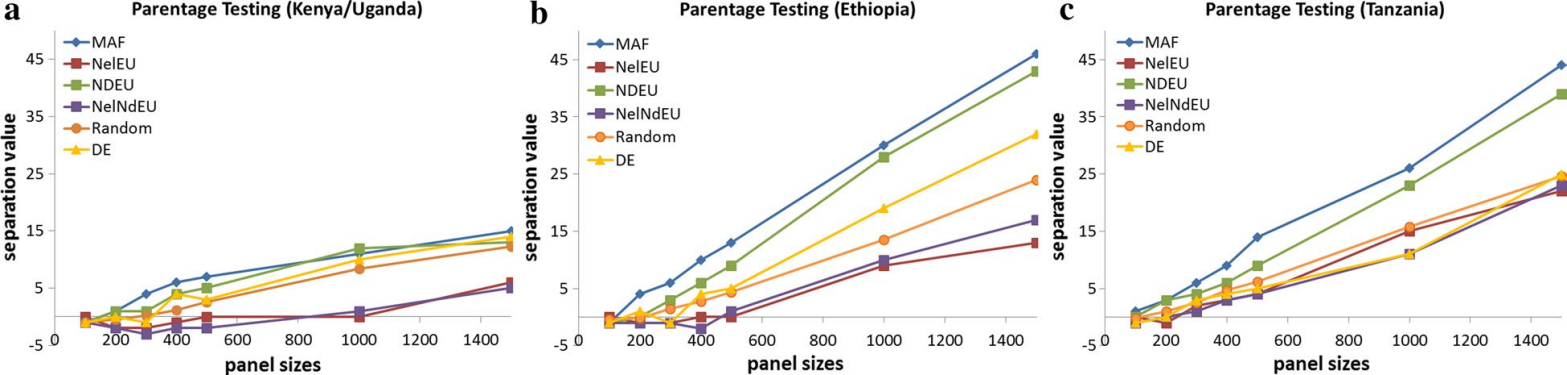
Parentage assignment for different panel sizes in three independent crossbred cattle populations (**a**) Kenya/Uganda, (**b**) Ethiopia, (**c**) Tanzania

The separation value (*sv*) provides a measure of the difference in *opH* in true parent–offspring relationships and other forms of relationships and in unrelated individuals. A *sv* lower than 0 indicates that the panel cannot reliably separate parent–offspring status from other relationships. SNPs with a high MAF have the highest probability of having *opH* between two unrelated individuals within a population under Hardy–Weinberg equilibrium. Thus, panels of SNPs that were selected for a high MAF in the crossbred population should perform best in assigning parentage using *opH* criteria. Our hypothesis was confirmed since SNP panels based on MAF achieved the highest *sv* in the Kenyan/Ugandan, Ethiopian, and Tanzanian datasets (Fig. 8a–c). However, none of the 100-SNP panels had a *sv* higher than 0. With 200 SNPs, only the panel of MAF-based SNPs had a positive *sv* in all three populations. As the number of SNPs in the panel increased, all methods used to select SNPs eventually achieved positive *sv*. Although the panel of MAF-based SNPs was based on allele frequencies in the Kenyan/Ugandan population, it performed well in all three crossbred populations.

The panel derived by the DE algorithm performed erratically and did not achieve positive *sv* in all three populations with less than 400 SNPs. Gondro et al. [27] reported positive *sv* for a 100-SNP panel derived by the DE algorithm in a Hanwoo cattle population. However, the Hanwoo cattle population was relatively small and all animals were part of parent–offspring pairs, which will tend to lead to higher *sv* than the much larger population of largely unrelated animals in which we tested the DE algorithm. For a larger crossbred sheep population, at least 400 SNPs were required to achieve a positive *sv* in both the discovery and validation dataset [27], which is consistent with our results.

Since *sv* is an integer variable it can be difficult for a DE algorithm to evolve to a higher *sv* once the panels of SNPs being evaluated by the algorithm all achieve the same *sv* and this may limit the ability of DE to find an optimum solution. The DE algorithm might perform better if it is initiated with prior knowledge on suitable SNP panels (e.g. panels of MAF-based SNPs, or SNP spacing restrictions) but it would still likely generate spuriously high *sv* values due to ascertainment bias.

The ISAG panel and the 50k-SNP chip yielded *sv* of 1 and 170, respectively, in the Kenyan/Ugandan dataset, 3 and 944, respectively, in the Ethiopian dataset, and 5 and 796, respectively, in the Tanzanian dataset.

The average *sv* of the randomly selected panels indicated that at least 300 SNPs are required to achieve a positive *sv*, which is in concordance with the findings of Strucken et al. [23], who reported that 340 randomly selected SNPs were needed for a positive *sv* in a composite cattle population.

We investigated whether the accuracy of the MAF-based panel was affected by breed composition. When using all 735k SNPs, dairy proportions of the animals in parent–offspring pairs ranged from 11 to 99%. Average *opH* counts for parent–offspring pairs with dairy proportions higher than 0.5 and those with dairy proportions lower than 0.5 were equal to 293 (SD = 93) and 420 (SD = 84), respectively. Although statistically highly significant (P < 0.0001), this difference is small since only a proportion of SNPs were tested with the SNP panels. MAF for animals with dairy proportions lower than 0.5 versus higher than 0.5 were virtually identical (0.2738 vs. 0.2734), and when using the MAF-based panel with either 200 or 400 SNPs, there was no significant correlation between *opH* and dairy proportion. Therefore, parentage assignment of the MAF panel is not expected to be affected by dairy proportion.

### Applications in the field

The NelNdEU panel was superior for the prediction of dairy proportions in all three populations: Kenyan/Ugandan, Ethiopian and Tanzanian. This panel was chosen based on allele frequencies in reference samples of Nelore, N’Dama, and *Bos taurus* dairy breeds, and no information from crossbred animals was used to select the SNPs. Thus, there is no ascertainment bias in the estimated accuracies with the NelNdEU panels using ADMIXTURE in our datasets. The 200-SNP panel provides a good compromise between a small number of SNPs while achieving high accuracy and the lowest absolute bias (r^2^ = 0.974 SE = 0.004; absolute bias = 0.031 using ADMIXTURE). In practice, not all of the 200 SNPs are available due to genotyping errors or to failure of some SNPs to work on a given assay platform. Therefore, we randomly selected 75 to 95% of the SNPs by simulating a genotyping failure of 5 to 25%. Accuracies of predicted dairy proportions remained high with an r^2^ of 0.965 and 0.975 when 75 and 95% of the genotypes were available, respectively. Thus, the NelNdEU 200-SNP panel should perform well in the field for the prediction of dairy proportions.

The MAF-based panel performed best for parentage assignment and achieved a positive *sv* of 1 with 200 SNPs. Similarly, we calculated the *sv* assuming a random genotyping failure of 5 to 25%. The *sv* was positive with 1 *opH* between true and false parentages. Thus, the MAF-based 200-SNP panel should perform well in the field for parentage assignment.

**Fig. 9:**
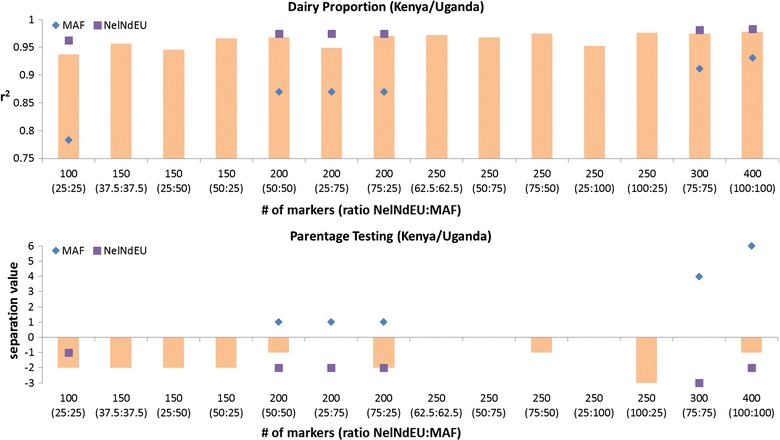
Accuracy (r^2^) of dairy proportion estimates and parentage assignment (*sv*) for combined panels. The *X-axis* shows the total number of SNPs in the panel and in brackets the percentage of the best SNPs chosen from each 200-SNP panel (NelNdEU:MAF). Symbols for MAF and NelNdEU show r^2^ and separation value (*sv*) for panels in separate evaluations

The NelNdEU panel performed poorly for parentage assignment whereas the MAF-based panel performed poorly for the prediction of breed composition. We explored the possibility of having a single, combined panel that performed well for both prediction of breed composition and parentage assignment. We tested panel sizes from 100 to 400 SNPs, and different combinations of SNPs from the NelNdEU and MAF-based panels. In each case, the best SNPs for each selection criterion (i.e. large allele frequency difference between NelNd and EU or highest MAF) were chosen from each panel. Using all 200 SNPs from each panel (i.e. 400 SNPs) resulted in an r^2^ of 0.978 (SE = 0.003; absolute bias = 0.027) for the prediction of breed proportion and a *sv* of −1. These results are slightly less good than those achieved by the NelNDEU and MAF-based 400-SNP panels (Fig. 9).

The combined panels performed relatively well for prediction of breed proportions, especially if more than 50% of SNPs were chosen from the NelNdEU panel. The combined panels did not achieve a positive *sv*, even if the majority of SNPs were chosen from the MAF panel. This is due to the fact that the 200-SNP panel chosen for high MAF is not performing well enough, i.e. the positive *sv* is not sufficiently high, to counteract the negative *sv* value of the NelNdEU panel. The 300 or 400 SNPs from the NelNdEU and MAF panels resulted in positive *sv* values. However, combining these to create a panel of 600 or 800 SNPs means doubling the number of SNPs. Therefore, we recommend using the 200 SNPs of the NelNdEU panel for prediction of breed proportions or the 200 SNPs of the MAF panel for parentage assignment.

The 1500 SNPs in the NelNdEU and MAF panels are provided in rank order of selection in Table S1 [see Additional file 9: Table S1], from which all the panels with smaller numbers of SNPs described in this paper can be reconstructed.

## Conclusions

For East African crossbred dairy cattle populations, it is possible to create SNP panels with as few as 200 SNPs that will result in accurate estimates of dairy proportions and panels of similar size but with different SNPs to assign parentage accurately. A single combined panel of 400 SNPs achieved sufficient accuracies for breed proportion prediction but was not able to assign parentages correctly. Results of the 200-SNP panels chosen independently for breed proportion prediction and parentage assignment indicate that these panels should be reliable for animals that are crossbred to a wide range of African indigenous breeds. However, they are not expected to perform well outside of Africa where indigenous breeds do not originate from ancient crosses between African *Bos taurus* and *Bos indicus* populations. Alternative panels based on SNPs that differentiate *Bos indicus* from European *Bos taurus* should perform well in countries where the indigenous base population is *Bos indicus*, such as in south Asia, but this needs to be tested.

## Additional files

**Additional file 1: Figure S1.** 3D-plots of principal components for reference, indigenous, and crossbred populations from Kenya/Uganda, Ethiopia, and Tanzania.

**Additional file 2: Figure S2.** Allele frequencies for SNP panels selected for the largest allele frequency differences of ancestral breeds, i.e. between Nelore or N’Dama and a weighted EU average in Nelore, N’Dama, or European populations.

**Additional file 3: Figure S3.** Allele frequencies for SNP panels in a crossbred cattle population (Ethiopia). Bold horizontal lines indicate the median and + indicates the mean.

**Additional file 4: Figure S4.** Allele frequencies for SNP panels in a crossbred cattle population (Tanzania). Bold horizontal lines indicate the median and + indicates the mean.

**Additional file 5: Figure S5.** Regression of estimates of dairy proportions (735k) on predictions of the NelNdEU 200-SNP panel.

**Additional file 6: Figure S6.** Regression of estimates of dairy proportions (735k) on predictions of the NelNdEU 400-SNP panel.

**Additional file 7: Figure S7.** Validation of dairy proportion estimates from one half of the Kenyan/Ugandan crossbreds (NelNdEU panel). (a) Validated in the other half of the Kenya/Uganda dataset. (b) Validated in independent populations from Ethiopia and Tanzania plus the second half of the Kenyan/Ugandan dataset.

**Additional file 8: Figure S8.** Accuracy (r^2^) of dairy proportion estimates and parentage assignment with increasing number of iterations.

**Additional file 9: Table S1.** Best 1500 SNPs for breed proportion estimation (NelNdEU) and parentage assignment (MAF). They are presented in rank order, from best to worst SNP.
