# Supplementary figures and images for: Genetic tests for estimating dairy breed proportion and parentage assignment in East African crossbred cattle
Source: Genet Sel Evol. 2017 Sep 12;49:67. doi: 10.1186/s12711-017-0342-1 (PMC5596489; doi:10.1186/s12711-017-0342-1)

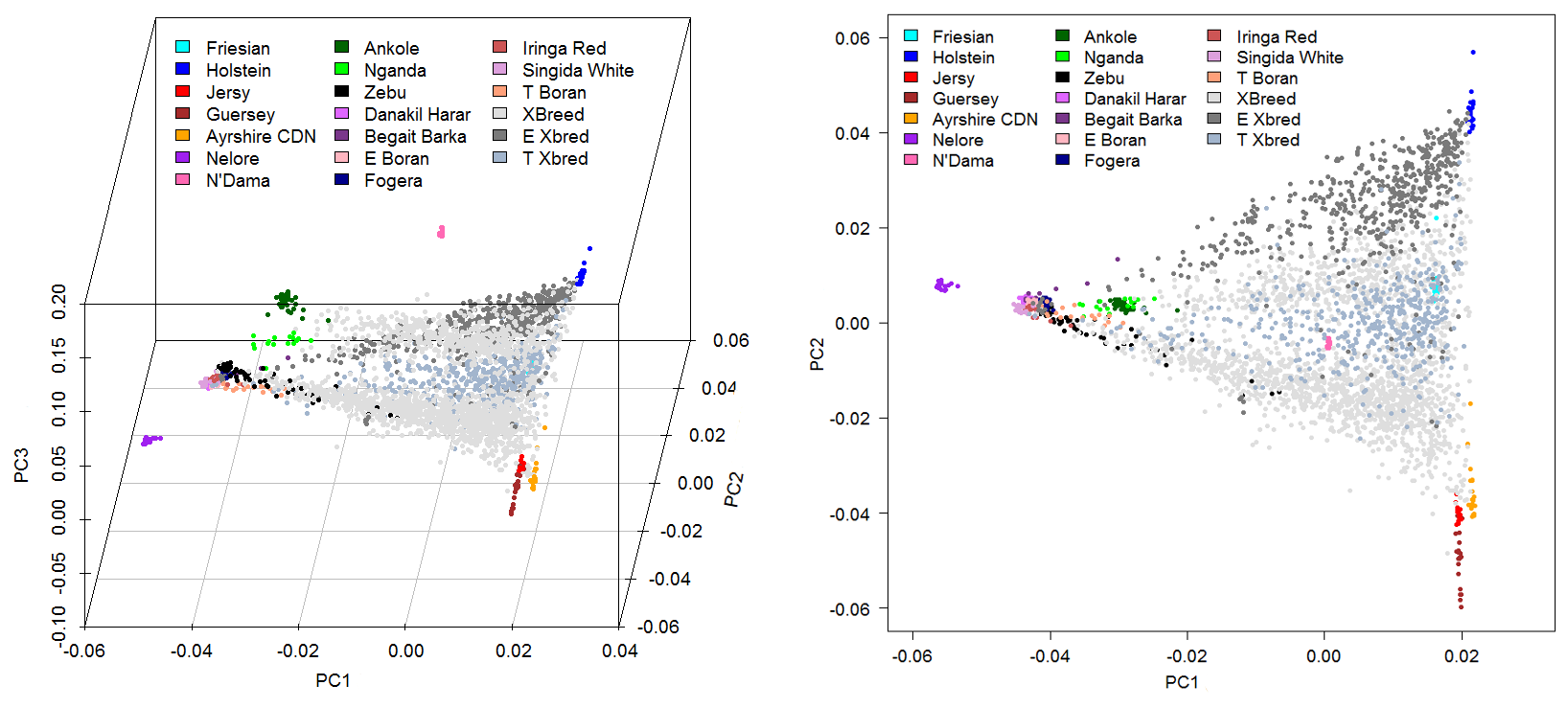

Supplement: Supplementary file 1 — Additional file 1: Figure S1. 3D-plots of principal components for reference, indigenous, and crossbred populations from Kenya/Uganda, Ethiopia, and Tanzania. [file 12711_2017_342_MOESM1_ESM.tiff]

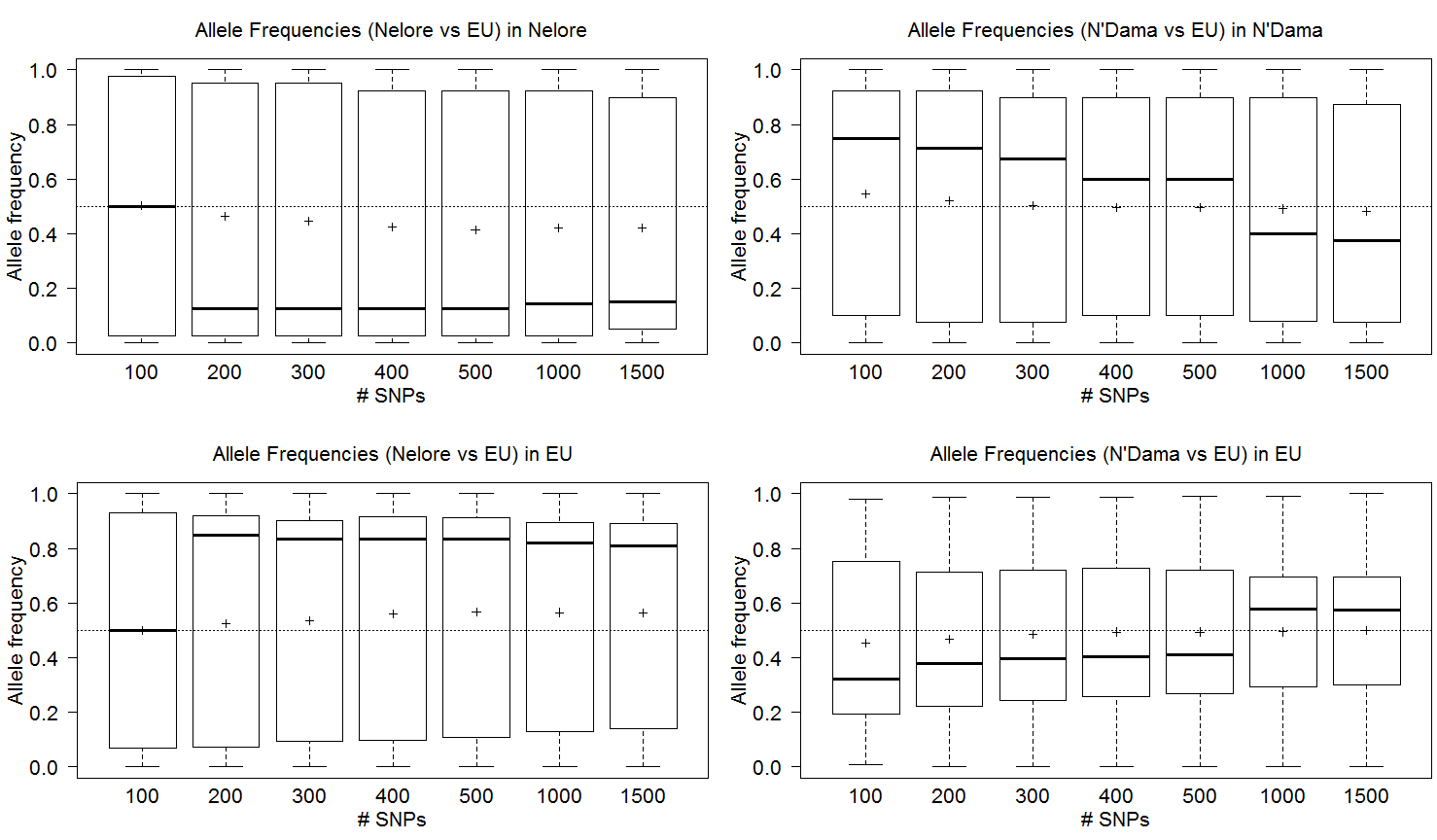

Supplement: Supplementary file 2 — Additional file 2: Figure S2. Allele frequencies for SNP panels selected for the largest allele frequency differences of ancestral breeds, i.e. between Nelore or N’Dama and a weighted EU average in Nelore, N’Dama, or European populations. [file 12711_2017_342_MOESM2_ESM.tiff]

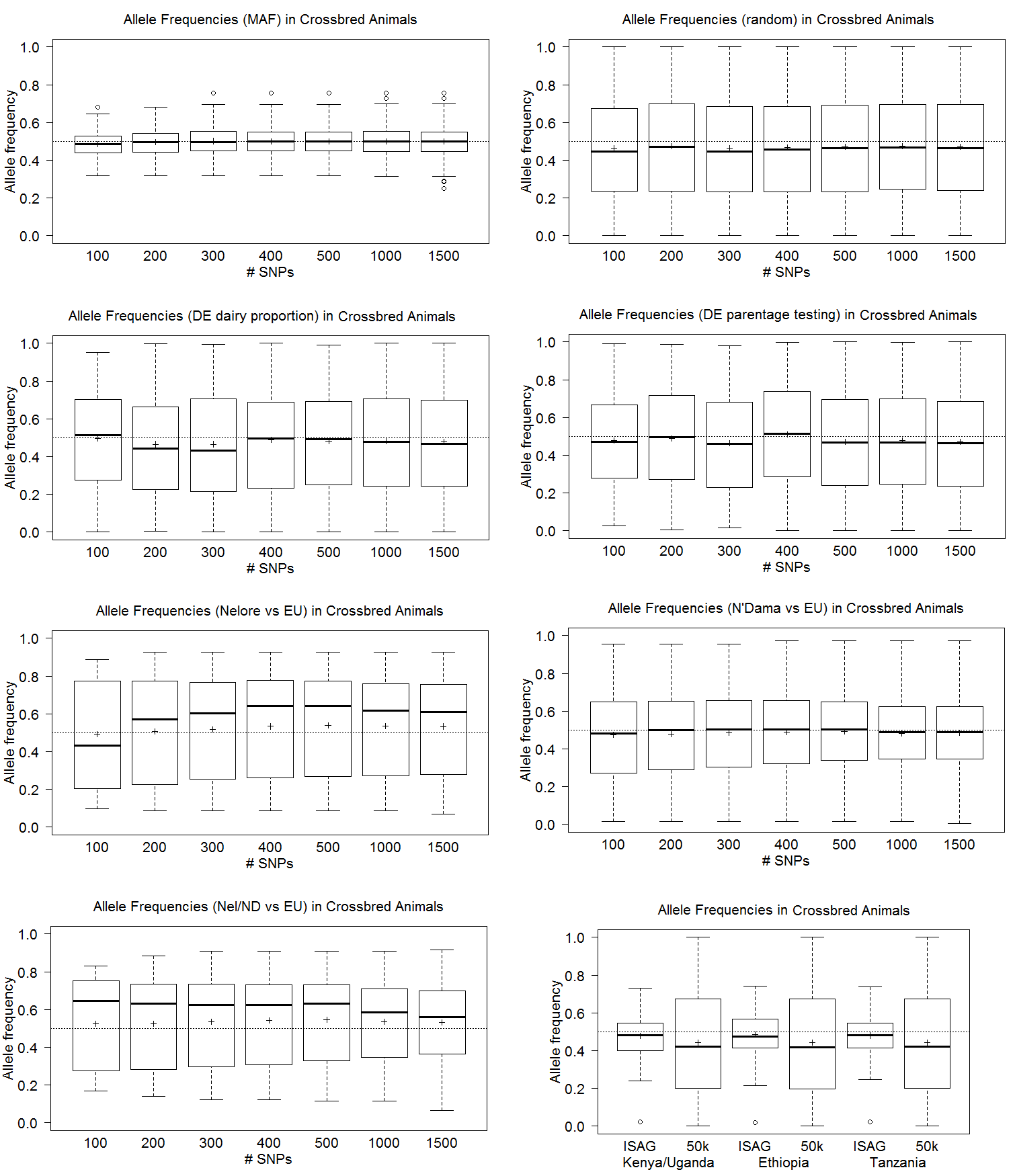

Supplement: Supplementary file 3 — Additional file 3: Figure S3. Allele frequencies for SNP panels in a crossbred cattle population (Ethiopia). Bold horizontal lines indicate the median and + indicates the mean. [file 12711_2017_342_MOESM3_ESM.tiff]

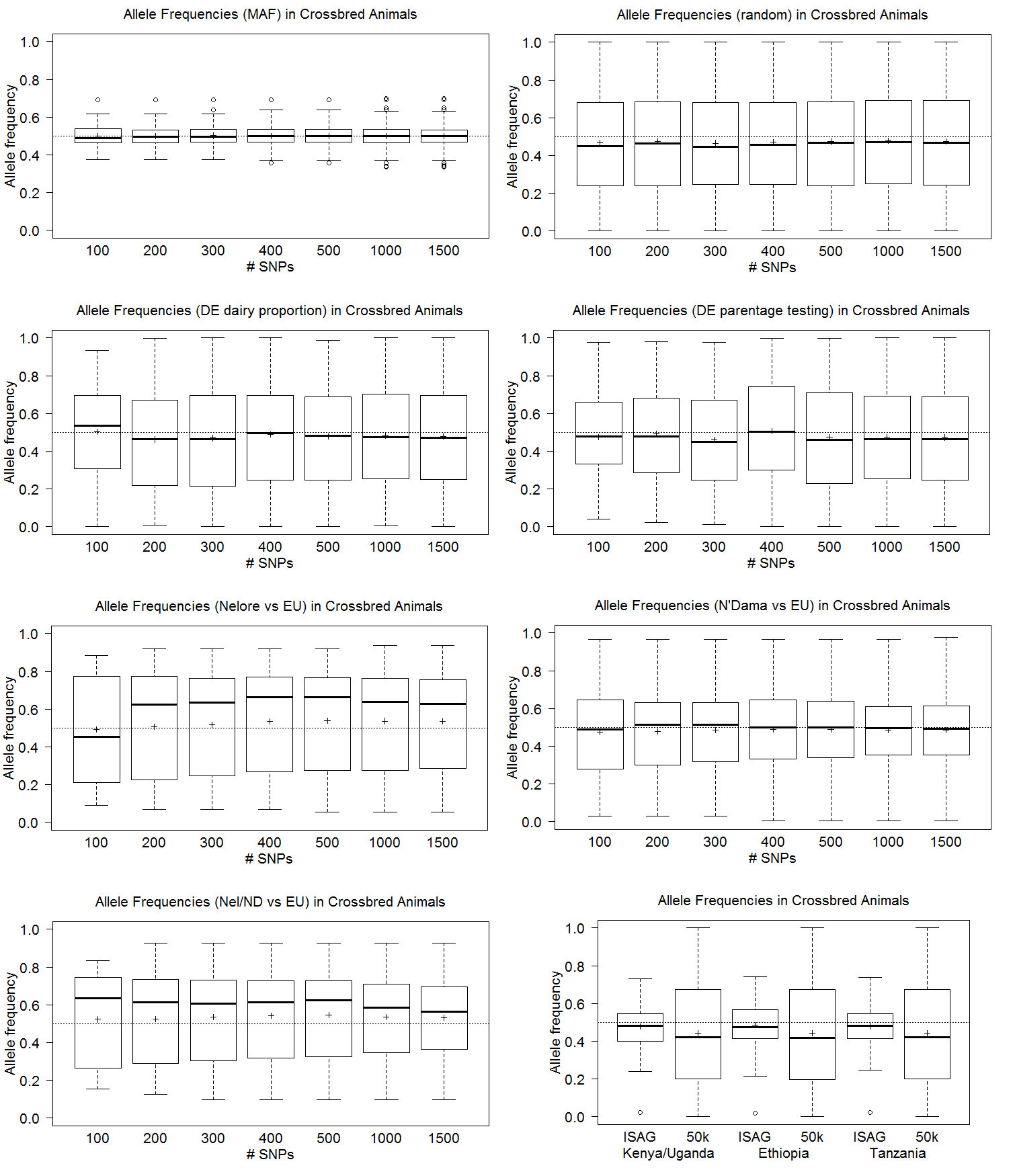

Supplement: Supplementary file 4 — Additional file 4: Figure S4. Allele frequencies for SNP panels in a crossbred cattle population (Tanzania). Bold horizontal lines indicate the median and + indicates the mean. [file 12711_2017_342_MOESM4_ESM.tiff]

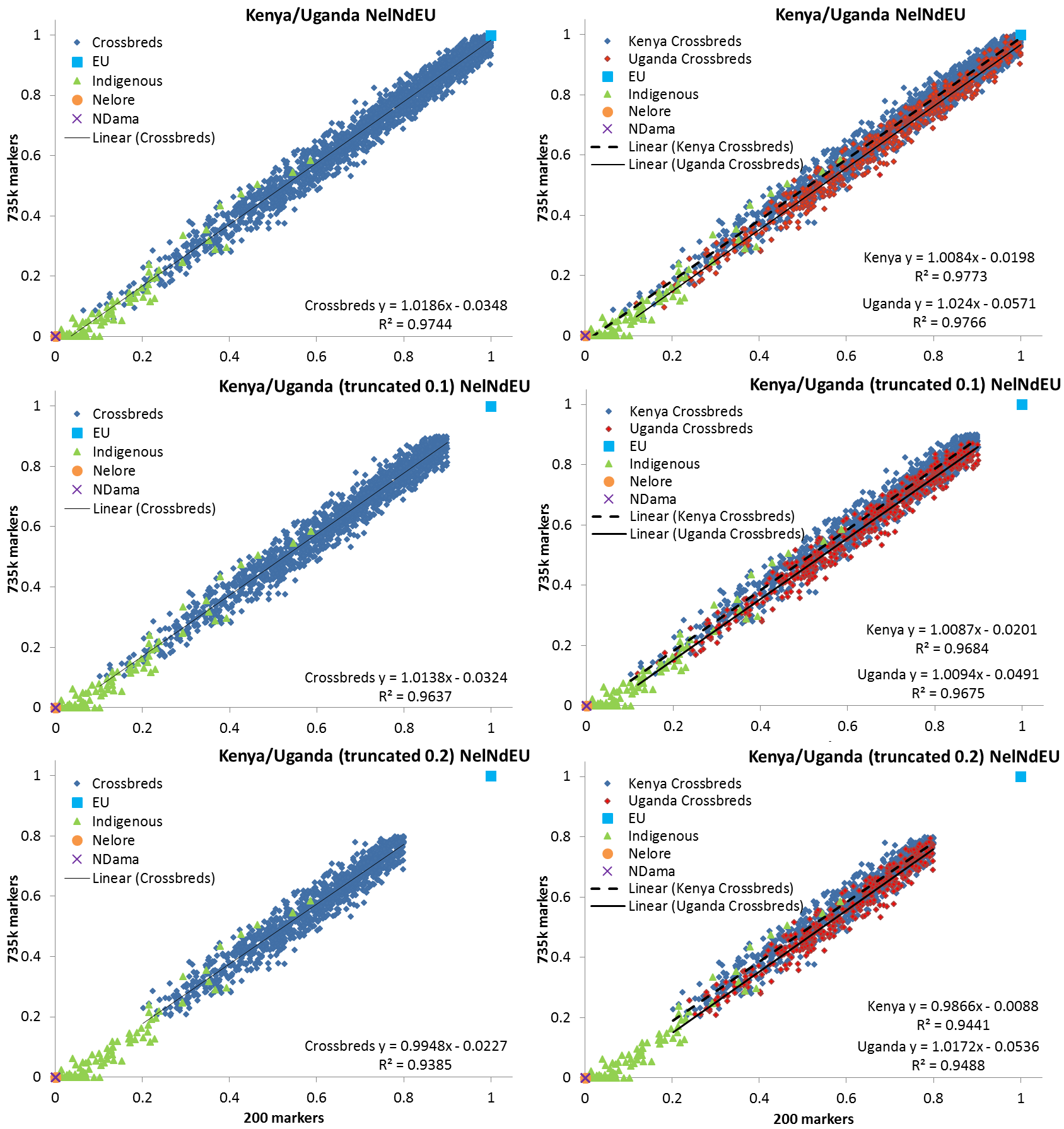

Supplement: Supplementary file 5 — Additional file 5: Figure S5. Regression of estimates of dairy proportions (735k) on predictions of the NelNdEU 200-SNP panel. [file 12711_2017_342_MOESM5_ESM.tiff]

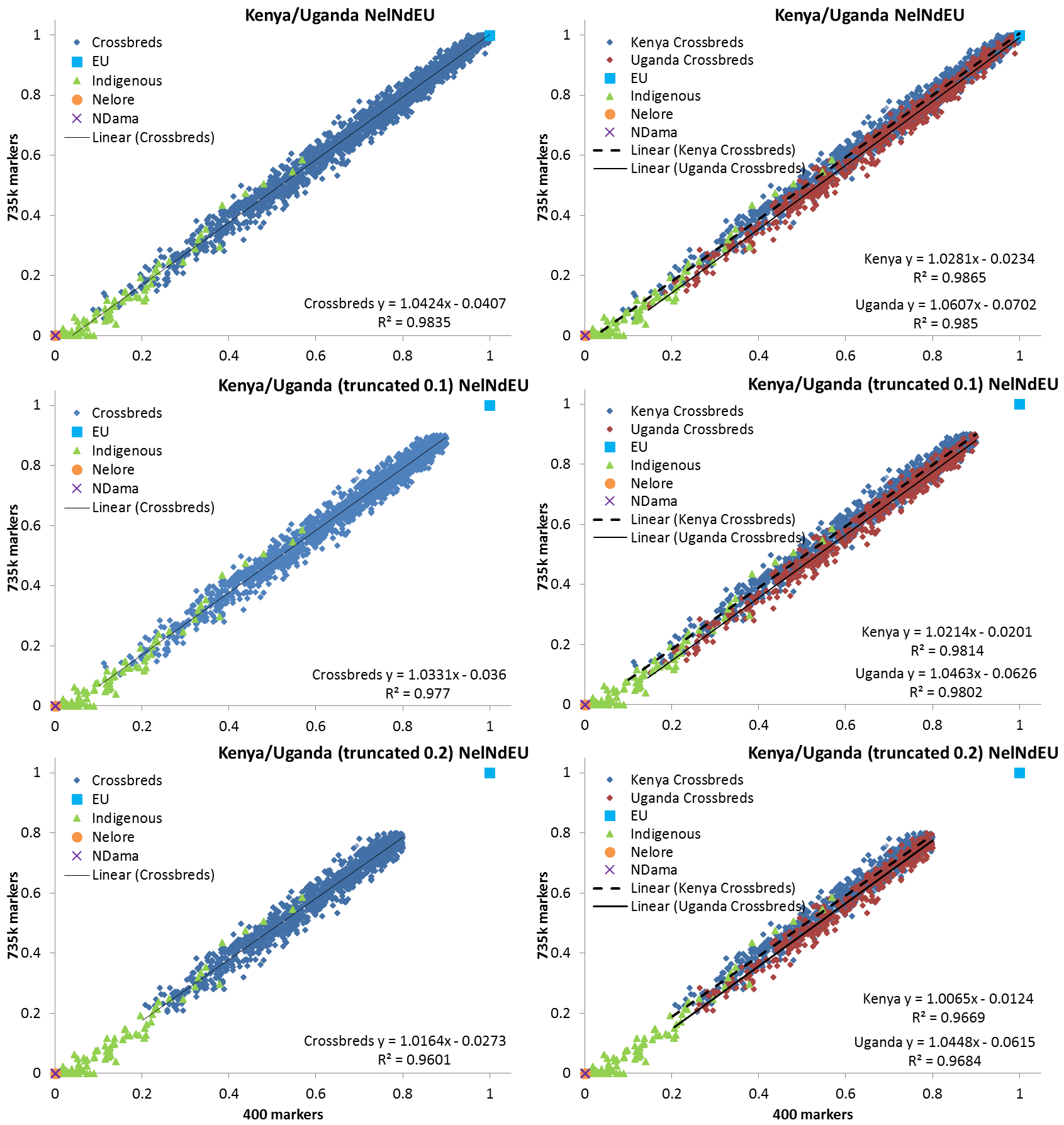

Supplement: Supplementary file 6 — Additional file 6: Figure S6. Regression of estimates of dairy proportions (735k) on predictions of the NelNdEU 400-SNP panel. [file 12711_2017_342_MOESM6_ESM.tiff]

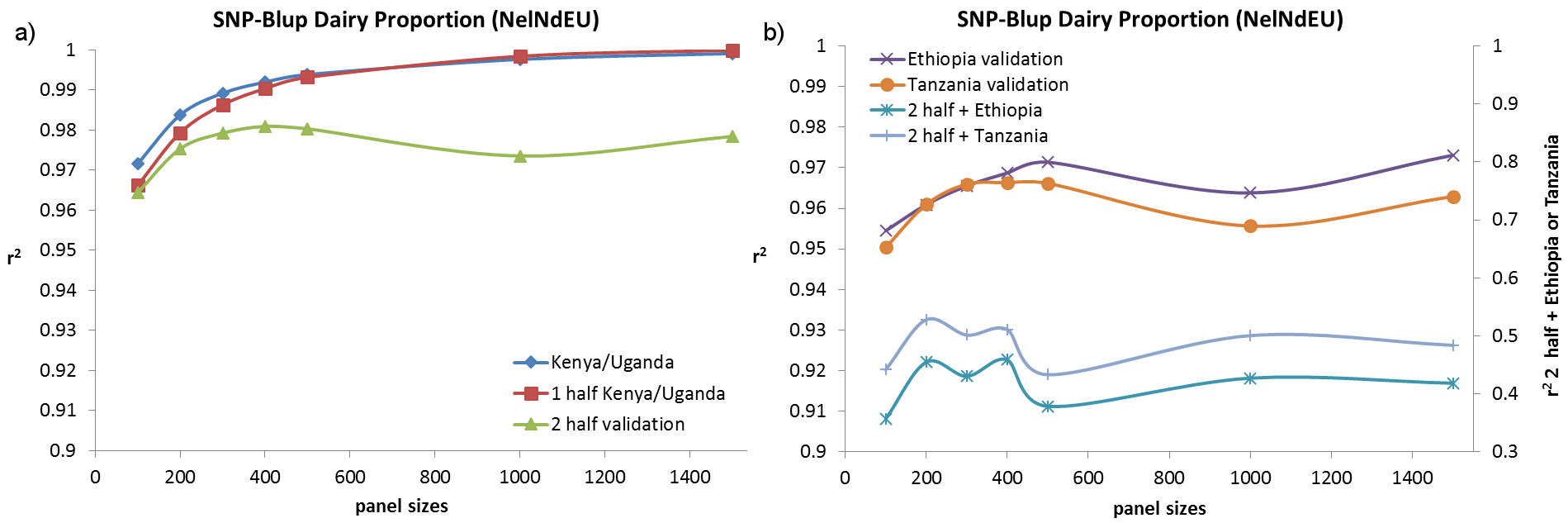

Supplement: Supplementary file 7 — Additional file 7: Figure S7. Validation of dairy proportion estimates from one half of the Kenyan/Ugandan crossbreds (NelNdEU panel). (a) Validated in the other half of the Kenya/Uganda dataset. (b) Validated in independent populations from Ethiopia and Tanzania plus the second half of the Kenyan/Ugandan dataset. [file 12711_2017_342_MOESM7_ESM.tiff]

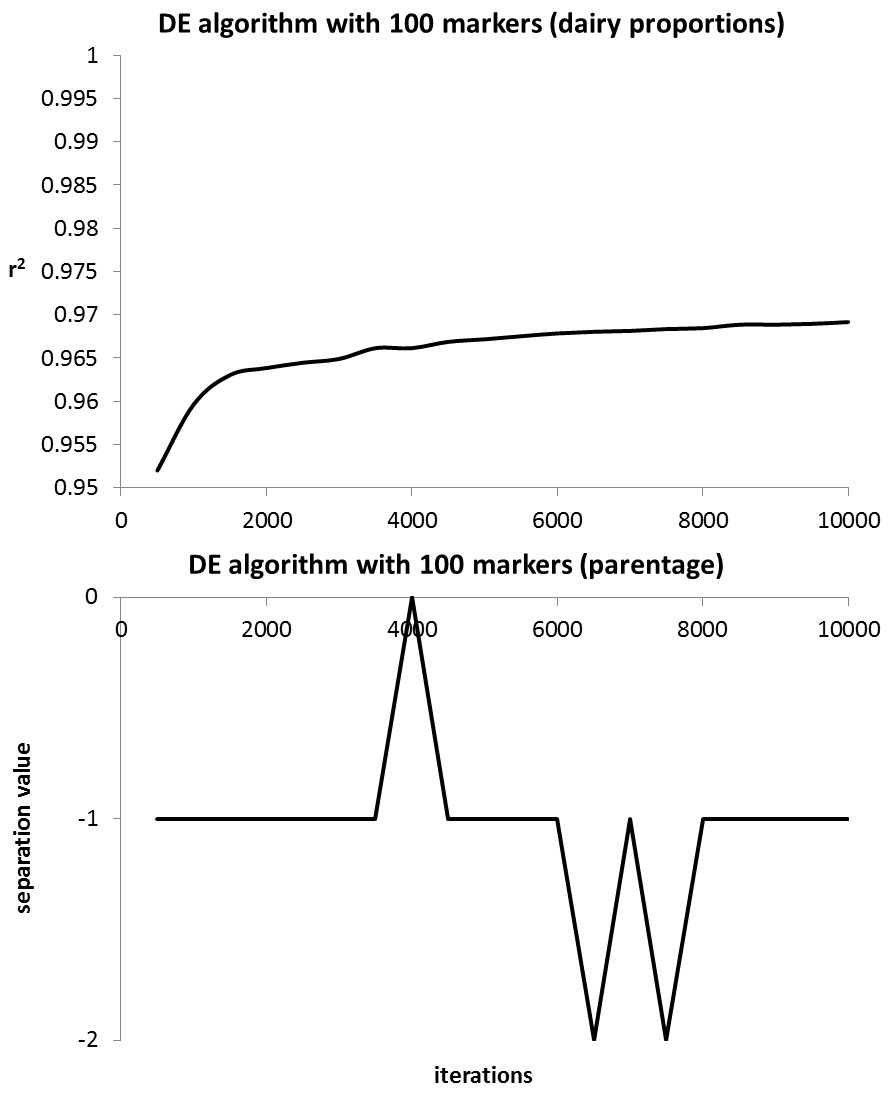

Supplement: Supplementary file 8 — Additional file 8: Figure S8. Accuracy (r2) of dairy proportion estimates and parentage assignment with increasing number of iterations. [file 12711_2017_342_MOESM8_ESM.tiff]
